# Students’ understanding of social determinants of health in a community-based curriculum: a general inductive approach for qualitative data analysis

**DOI:** 10.1186/s12909-020-02391-z

**Published:** 2020-11-25

**Authors:** Sachiko Ozone, Junji Haruta, Ayumi Takayashiki, Takami Maeno, Tetsuhiro Maeno

**Affiliations:** 1 Department of Family Medicine, General Practice and Community Health, Faculty of Medicine, University of Tsukuba, 1-1-1 Tennodai, Tsukuba, Ibaraki, 305-8575 Japan; 2 Department of Primary Care and Medical Education, Faculty of Medicine, University of Tsukuba, 1-1-1 Tennodai, Tsukuba, Ibaraki, 305-8575 Japan; 3 Medical Education Center, School of Medicine, Keio University, 35 Shinanomachi, Shinjuku, Tokyo, 160-8582 Japan

**Keywords:** Community based education, Primary care education, Social determinants of health

## Abstract

**Background:**

A community-based medical education (CBME) curriculum may provide opportunities to learn about the social determinants of health (SDH) by encouraging reflection on context, but the categories that students can learn about and their level of reflection are unclear. We aimed to analyze medical students’ understanding and level of reflection about SDH in a CBME curriculum.

**Methods:**

Study design: General inductive approach for qualitative data analysis.

Education Program: All 5th-year and 6th-year medical students at the University of Tsukuba School of Medicine in Japan who completed a mandatory 4-week clinical clerkship in general medicine and primary care during October 2018 and May 2019 were included. The curriculum included 3 weeks of rotations in community clinics and hospitals in suburban and rural areas of Ibaraki Prefecture. On the first day, students learned about SDH through a lecture and a group activity. As an SDH assignment, they were instructed to prepare a structural case description using the Solid Facts framework based on encounters during the curriculum. On the final day, they submitted the structural reflection report.

Analysis: Content analysis was based on the Solid Facts framework. Levels of reflection were categorized as reflective, analytical, or descriptive.

**Results:**

We analyzed 113 SDH case descriptions and 118 reports. On the SDH assignments, the students frequently reported on social support (85%), stress (75%), and food (58%), but less frequently on early life (15%), unemployment (14%), and social gradient (6%). Of the 118 reports, 2 were reflective, 9 were analytical, and 36 were descriptive. The others were not evaluable.

**Conclusions:**

The CBME curriculum enabled medical students to understand the factors of SDH to some extent. Further work is needed to deepen their levels of reflection.

**Supplementary Information:**

The online version contains supplementary material available at 10.1186/s12909-020-02391-z.

## Background

Biological factors are only some of the factors that affect health. Social factors also affect an individual’s health. Various social conditions that could affect an individual’s health, such as socioeconomic conditions, poverty, education opportunities, employment status, and working environment, are defined as social determinants of health (SDH). SDH are most responsible for health inequities [1]. An approach involving biological factors alone may have limited effect on improving an individual’s health.

Educating healthcare professionals on SDH may be one way to affect SDH and health inequities. The role of health advocate is considered a key competency of physicians in the CanMEDS framework by the Royal College of Physicians and Surgeons of Canada [2]. It states that physicians should be able to respond to the needs of patients, communities, or populations by advocating with them beyond the clinical environment. In Japan, the ability to outline SDH was introduced to the model core curriculum for medical students in 2017 [3].

The National Academies of Science, Engineering, and Medicine provides a framework for lifelong learning for health professionals in understanding SDH [4]. In this framework, lifelong learning about SDH is built around three domains: education, community, and organization. Concerning learning within the community, community-based education curriculum aim to be educationally relevant to community needs by providing education within the community [5]. Since community-based medical education (CBME) curriculums are commonly provided by medical schools, it may harmonize with SDH education for medical students.

Although SDH education for medical students is claimed to be necessary, the optimal teaching and evaluation methods are unclear. According to a review article about undergraduate medical education on SDH [6], the most common type is experiential learning in the form of community-based or clinic-based learning, with the majority of the assessments being subjective and self-reported. Although critical reflection is valuable for transforming learner attitudes [7], few research studies have assessed students reflectively. In addition, the readiness of medical students to learn about SDH is unclear. Moreover, adding new content to undergraduate medical education may often be difficult because in many cases the curriculum already includes much content. Utilizing an existing CBME curriculum may be one solution for SDH education to medical students. The objective of this study was to analyze medical students’ understanding and level of reflection about SDH-related experiences in a CBME curriculum.

## Methods

### Study design

The design of this study was a general inductive approach for qualitative data analysis. It evaluated SDH worksheets and reports of medical students who completed SDH-related assignments in a CBME curriculum.

The general inductive approach is a systematic procedure for analyzing qualitative data in which the analysis is likely to be guided by specific evaluation objectives. It aims to allow research findings to emerge from frequent, dominant, or significant themes inherent in the raw data, without restraints imposed by structured methodologies [8].

### Population and settings

The study population consisted of fifth-year and sixth-year medical students in the University of Tsukuba School of Medicine who completed a mandatory 4-week clinical clerkship in the CBME curriculum between September 2018 and May 2019.

### CBME curriculum

The 4-week CBME curriculum is a part of the introduction to medicine course. The introduction to medicine course is a 6-year curriculum for learning about the basics essential for healthcare professionals, such as medical ethics, primary care, health promotion, professionalism, and interprofessional collaboration. The CBME curriculum is the clinical clerkship component of the introduction to medicine course. Within the introduction to medicine course, some assignments required students to write reflective reports, but students were not offered training opportunities dedicated to reflective writing.

The aims of the CBME curriculum were to: 1) understand the expertise of family physicians who provide appropriate medical care in various clinical settings, 2) understand the health issues of citizens, patients, and families from the perspective of the local healthcare system, and 3) acquire clinical reasoning skills.

Every 4 weeks, 15 to 17 students participate in the rotation. They spend 1 week in community-based settings, 1–2 weeks in community clinics or small hospitals, 0–1 week in community hospitals, and 1 week in the family medicine department of the university hospital. All settings were located in sub-urban or rural areas of Ibaraki Prefecture, Japan. The first and last days of the 4-week curriculum included meeting at the university hospital for orientation and summary. During the rotation, students experienced outpatient medical interviews and reflection, participated in home visits for medical and nursing care, provided health promotion classes to citizens or elementary or junior high school students, and experienced community diagnosis at the various settings. In community diagnosis, students collected both quantitative and qualitative data through observations, interviews, and retrieval of local data. The curriculum was organized by faculty members from the Department of Family Medicine at the University of Tsukuba School of Medicine and supported by physicians and other healthcare professionals and citizens in the community. Many field visits provided the opportunity for students and faculty members to reflect on what students had learned each week on the last day of their 1–2-week rotation. The students were evaluated based on their performance in each setting and the final report they write and submit on the final summary day.

### SDH program (Fig. 1)

During the orientation on the first day, students were given a case-based lecture on SDH and an SDH assignment worksheet (Supplementary Table 1) to complete during the 4-week clerkship. The SDH assignment sheet asked students to “choose a patient or a family that you encounter during the 4-week rotation, collect information, and consider possible background factors that may be affecting the health of the patient.” The Solid Facts 2nd edition [9] from the World Health Organization was provided as reference material. It lists ten factors related to SDH: the social gradient, stress, early life, social exclusion, work, unemployment, social support, addiction, food, and transportation. Students were assigned to present their cases in small groups on the final day.

**Fig. 1:**
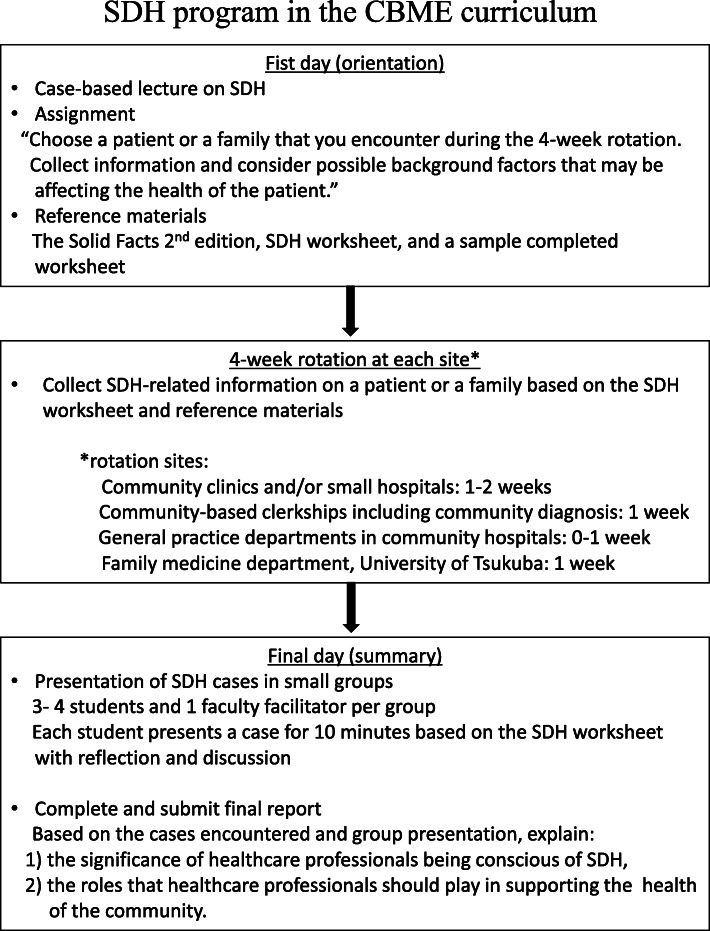
Overview of the social determinants of health program in the community-based medical education program at the University of Tsukuba School of Medicine

On the final day, students presented their SDH cases in a small discussion group with one faculty member acting as a facilitator for each group. Students were assigned a final report about the CBME curriculum. SDH were one topic for the report. The assignment asked students to explain the “significance of healthcare professionals becoming conscious of SDH” and “roles that healthcare professionals should play to support the health of the community.” The students were provided a rubric for how the final report will be graded (Supplementary Table 2).

The faculty members in each field supported the students as needed during the CBME program in the community. We provided several faculty development (FD) sessions before and during the SDH program. The FD sessions were held to help faculty members understand the significance, purpose, and method of SDH education within the CBME program and to improve how they provide feedback as facilitators during the group presentation on the final day.

Transformative learning [10] was the key learning theory that formed the basis of this program. Based on this theoretical framework, we planned to help medical students with a biomedical perspective gain a socioeconomic systems perspective by developing an educational program that emphasizes critical reflection and review of complex health care systems [4].

### Analysis

SDH worksheets were evaluated by content analysis based on the Solid Facts 2nd edition framework. SO evaluated the SDH worksheet and the results were verified by the other co-authors. Levels of reflection in the final reports were categorized as descriptive, analytical, or reflective, referring to the style of academic writing according to the University of Reading [11]. Level of reflection was measured as an indicator to evaluate the effect of the curriculum on the students’ transformative learning. A report that explained the case descriptively according to the SDH framework but lacked integration of the factors was categorized as descriptive. If the report integrated SDH factors, it was categorized as analytical. A report that further reflected on one’s own ideas about SDH was categorized as reflective. Reports that did not fall into any of these categories were categorized as unable to be evaluated. SO and JH independently assessed the levels of reflection and all researchers discussed and agreed with the results of the analysis. For reports where authors had different categorizations, the authors discussed the reasons for the categorization and level of reflection and reached consensus.

### Ethical approval

This study was approved by the University of Tsukuba medical ethics board (No. 2676).

## Results

During the study period, 118 medical students attended the program, of whom 35 students (29.7%) were female. A total of 113 SDH worksheets and 118 reports were analyzed.

**Fig. 2:**
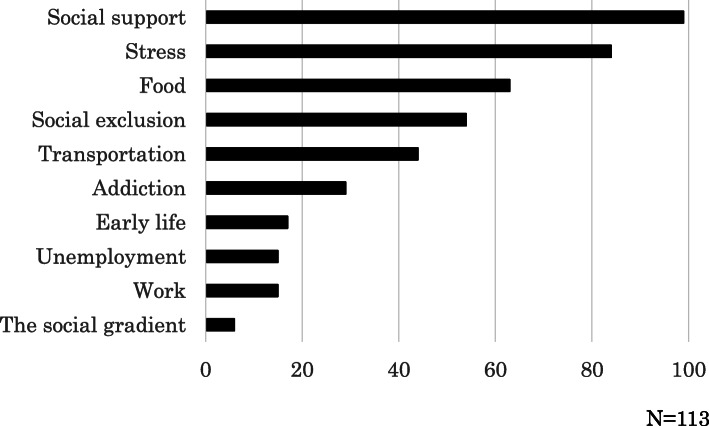
Number of students who listed each factor in the Solid Facts 2nd edition in the social determinants of health assignments

In the SDH worksheet, the students most frequently reported on social support; 99 students reporting on this SDH (Fig. 2). Students reported on relationships with family, friends, neighbors, or care staff as sources of social support for the patient. Other frequently reported topics included stress (84 students), food (63 students), social exclusion (54 students), and transportation (44 students). Stress was frequently reported as related to illness by the patient or the family or as part of relationships with the family members. Social exclusion was reported in the context of poverty, exclusion due to illness, or being a welfare recipient. On the other hand, early life (17 students), work and unemployment (14 students), and social gradient (6 students) were less commonly reported. The students reported on an average of 3.77 factors in the Solid Facts framework on the SDH worksheet; 75.2% of students reported on three or more factors. Table 1 shows examples of the experiences that students reported as related to SDH.

**Table 1:** Examples of SDH-related experiences reported in the SDH worksheet

	Examples of SDH-related experiences reported
The social gradient	Underpopulated area, lack of medical institutions, difficulty in getting a stable job due to illness
Stress	Illness, illness in a family member, relationship with the family (good or bad), lack of hobbies, loneliness, moving
Early life	Education, parents’ divorce, parents’ anxiety about parenting
Social exclusion	Poverty, medical expenses, exclusion due to illness, being a social welfare recipient or pensioner
Work	Stress or bullying at work, manual labor, shift work
Unemployment	Unemployed due to illness, unemployment due to earthquake
Social support	Relationships with family, friends, community, or nursing staff, long-term care services
Addiction	Alcohol, smoking, gambling
Food	Dependence on instant foods and convenience store meals, eating salty foods, growing fresh vegetables, cannot go shopping on their own
Transportation	Returned the driver’s license, no driver’s license, lack of public transportation, dependence on others for transportation

Legends: Students reported underpopulated area and lack of medical institution as the social gradient, patient or family’s illness as stress, relationship with family and community as social support, and lack of transportation as transportation issues related to SDH. Abbreviations: *SDH* social determinants of health

Of the 118 reports, 36 reports were categorized as descriptive, 9 reports as analytical, and 2 reports as reflective. The descriptive reports explained the current conditions of the patient using the SDH framework but lacked integration of the factors or comprehensiveness. (Table 2)

*“The diabetic patient I was in charge had less knowledge about diabetes (social disparity), liked thicker flavors, ate a lot of rice (food), had a poor work environment (work), and had worsening diabetes. I felt it was necessary to provide appropriate information to each patient.”*

The analytical reports explained SDH mainly in a single patient, discussed multiple SDH factors, and focused on upstream factors as solutions.

*“A woman in her forties who handles all housework alone was suspected of having a relapse of depression. Approaching only the current problems such as the lack of sleep and the burden of housework does not lead to a solution. The essential approach is to consider what is the cause.”*

The reports categorized as reflective explained and compared multiple patients encountered during the curriculum, discussed the relationship between SDH factors, and explained thoughts and ideas on the roles of healthcare professionals in decreasing health inequity.

*“Some people live in a dirty house, have loans, and can't pay for electricity. Others live in a clean house, and their families can clean their rooms every day and they get nutritious meals. The social disparities, stress, unemployment, food, childhood, etc. that we considered on this worksheet are all closely interrelated in the flow of individuals. Sharing appropriate knowledge will help each other in the community and improve overall health.”*

The remaining 71 reports were considered unable to be evaluated because they were not descriptive, analytical, or reflective. Most did not describe the relationship between one’s experience regarding SDH and their impact on health. Table 2 shows some examples of reports categorized as analytical or reflective.

**Table 2:** Examples of reports that are evaluated as descriptive, analytical, reflective, and unable to evaluate

Level of reflection	Report content
Descriptive	The diabetic patient I was in charge had less knowledge about diabetes (social disparity), liked thicker flavors, ate a lot of rice (food), had a poor work environment (work), and had worsening diabetes. As a healthcare professional, I felt it was necessary to provide appropriate information for each patient.
	Due to large social disparities (low income, living on a pension), some people will not be able to go to the hospital and will be left alone when sick or injured, even if they need medical care. There are many elderly people in areas where transportation systems are not maintained, such as mountainous areas, but they cannot drive or afford to use a taxi. In areas with many elderly people, healthcare professionals need to visit patient homes and understand their living conditions and their relationships with family members.
Analytical	A patient with hypertension and renal insufficiency had such poor compliance that dialysis was indicated but was not introduced. The patient was discharged home with a life expectancy of several weeks to months. Due to a severely limited financial situation, there were no choices for nursing homes or nursing care. The personality of the patient was too emotionally unstable, so all family members denied support. These various social determinants not only affected the patients themselves, but are also shared by families that live together. They are intertwined in complex ways and contributes to health to illness.
	There were many single male workers who were mainly engaged in manual labor. Drinking and smoking were common habits. Their lifestyles were irregular in a workplace with three work shifts. With this background in mind, we can see why an individual’s eating habits were disrupted and how we can improve their lives. Approach to these determinants can break the roots of a vicious cycle that might include being transported to a hospital repeatedly because of continuing the same lifestyle.
	A woman in her forties who handled all housework alone was suspected of having a relapse of depression. Approaching only the current problems such as the lack of sleep and burden of housework does not lead to a solution. The essential approach is to consider what is the cause. Why was she in a situation where she had to do both housework and work? What was the economic situation? Was there a relationship with the community? Taking an even more upstream perspective is especially important when you cannot solve the problem or you cannot expect a solution.
Reflective	The social disparity was very impressive. Some people live in a dirty house, have loans, or can’t pay for electricity. Others live in a clean house and their families can clean their rooms every day and they get nutritious meals. The differences in clothing, eating, and living are greatly related to quality of life and, in turn, to patient health. The social disparities, stress, unemployment, food, childhood, etc. that we considered in this worksheet are all closely related and interrelated in the flow of individuals. Poor environments increase the risk of lifestyle-related and infectious diseases and poor nutritional balance can lead to reduced immune function and ill health. One role that healthcare professionals should play in a community is to provide necessary health education. There are many places in the area where there is a system for cooperating with people around me, but sharing appropriate knowledge will help each other in the area and improve overall health.
	Symptoms of an outpatient were related to stress (tired from family’s care, work fatigue, etc.), so we considered how to relieve stress. Tobacco use and gambling addiction were caused by poor work performance, unemployment, and poor family conditions. When unemployment, childhood events, family environment, etc. are related to health status, some people may not want to talk about their situation or want interference. Some patients may not have been able to speak out, but still I think it’s important to listen. The physician I met decided to become an occupational physician because many of the patients he met in the emergency department had health problems caused by their lifestyle, and he wanted to improve from there. In some cases, he was appreciated for conducting smoking cessation programs in the workplace because the results of medical examinations improved. As a healthcare professional, I thought that this can be done in a regional framework. I can understand the characteristics related to local health in advance and give lectures and advice.
Unable to evaluate	From the case of a paralyzed patient living in a dirty house, I learned that even with the same disease, the living environment could be abnormal beyond my imagination. In such a living environment, there are more diseases such as infectious diseases, so I felt it was meaningful for medical professionals to be aware of social determinants of health. I think the most important thing in the region is to improve medical facilities and build human relationships with local people.
	There was a patient who was discharged early because of cigarettes and gambling. Cigarettes and gambling appeared to have a negative effect on health and should be stopped. However, for that patient, the place where he gambled was important for social relationships; without it, dementia may occur. I thought that focusing on SDH would enable us to provide medical care for patients that considered more than one aspect. The role of healthcare professionals in the community is not only to treat the disease, but also to consider and help ensure happiness for the patient.

Legends: The descriptive reports explained the current condition of the patient using the SDH framework but lacked integration of the factors. The analytical reports explained SDH discussed multiple SDH factors mainly in a single patient and focused on upstream factors. The reflective reports explained and compared multiple patients and discussed the relationship between SDH factors and explained ideas on the role of healthcare professionals in decreasing health inequity. Abbreviations: *SDH* social determinants of health

## Discussion

Through an SDH education program in a CBME curriculum, students were able to focus on some factors listed in the Solid Facts framework. Some students were able to reflect on their SDH-related experiences at an analytical to reflective level.

SDH factors that are more related to medical care or can be addressed at the present time, e.g., social support, food, social exclusion, or transport, seemed to have received more attention. Students often shadowed physicians, nurses, or care staff providing home-visit care. These opportunities may have enabled them to see the “real life” of the patients, including social support, social exclusion, and transportation.

Although students focused on a variety of SDH factors, 75% of the students were able to list three or more SDH factors. The Solid Facts served as a practical framework for focusing students on SDH during the course. On the other hand, some SDH factors such as early life and social gradient seemed to be difficult to understand. This may have been due to relatively short length of the program (4 weeks), difficulty in assessing the relationship between the population’s experience and its impact on health over time, and difficulty in understanding SDH factors that are not readily apparent. Work and unemployment may have been mentioned less often because of the characteristics of patients encountered during the program; more patients were elderly or retired individuals as opposed to younger adults.

Only a few students were able to describe their SDH-related experiences reflectively. Reviews on reflective writing in healthcare education report that students tend to reflect on descriptive levels [12, 13] and deeper levels of reflection appear to be more difficult to achieve [14]. The ability to reflect seems to vary across individuals, but skills may be developed over time and with practice [14]. The comprehensive model of SDH may have been difficult to understand for medical students who have been mainly trained based on the medical model. They may not have had enough experience in community-based settings to deepen their reflection in the community context. Further FD may have been necessary to enable students to understand the comprehensive model of SDH. Furthermore, students may not have had enough experience in reflective writing itself that their readiness to reflect was inadequate. This may be a challenge for undergraduate medical education as well as SDH education in a CBME curriculum. Although the majority of reports were categorized as not reflective, many reports showed a tendency to focus beyond the medical model and shift toward the biopsychosocial model. SDH education in a CBME curriculum provides an opportunity for students to assess and reflect on a paradigm that is different from the medical model.

The strength of this program was that we showed the framework for about learning SDH to students by providing them with the Solid Facts. Reflective thinking in students is reported to be enhanced with guidelines and feedback [13], and students were able to depict SDH factors when guided by the framework. In some cases, however, the framework encouraged students to find cases that best match the framework. The other strength was that we provided an opportunity for students to share their experiences on cases involving SDH in a group discussion with one faculty member as the facilitator for each group. Mentoring and group discussion have also been reported to be related to the development of reflection [14].

On the other hand, the program also needed some improvement. First, the goals of the program need to take into account the relatively short duration of the program. Some of the solutions may be to set the goal of this program as understanding the basics of SDH and to connect them to further professional learning because SDH education is an ongoing process that involves professional practice [4]. Others may be to provide additional opportunities to experience diverse cases involving SDH within and beyond this curriculum, which may improve students’ readiness for SDH education and enhance longitudinal learning about SDH, further efforts are needed to deepen the level of reflection. Development of reflection is reportedly related to having a supportive environment, authentic context, mentoring, and perception of relevance [14]. Reflection of the faculty on the process of teaching reflective learning may also improve the process [7]. Although we provided FD sessions before and during the SDH program, there may have been differences in how individual faculty members understood and provided SDH education. Improvements in FD sessions for faculty so that they are able to support students during the SDH program may deepen students’ understanding of SDH and contribute to higher levels of reflection.

There were some limitations to this study. The study subjects were from a one-year program at a single medical school. In addition, the CBME setting was limited to one region of Japan, mainly in sub-urban or rural areas. No metropolitan or isolated island settings were included. However, many CBME programs are provided by medical schools worldwide in similar settings [15, 16]. We believe that our results could be applied to many undergraduate CBME programs that aim to provide SDH education. The SDH worksheet was intended for students to find a case during the curriculum and to present it to the group on the final day. Thus, level of reflection could not be assessed using the SDH worksheet. The SDH worksheet and the report were not linked on the individual student level, which precluded evaluation of how the learning process of individual students proceeded. These are issues for further research.

## Conclusion

An SDH education program within a CBME curriculum for medical students provided an opportunity for students to understand some SDH factors. Future approaches that include faculty development and multidimensional programs may be needed for SDH education to be more reflective.

## Supplementary Information

**Additional file 1.**

**Additional file 2.**

**Additional file 3.**

## Data Availability

All data analyzed during the current study are available from the corresponding author on reasonable request.

## Abbreviations

SDH: Social determinants of health
CBME: Community-based medical education
