# Supplementary material for: Students’ understanding of social determinants of health in a community-based curriculum: a general inductive approach for qualitative data analysis
Source: BMC Med Educ. 2020 Nov 25;20:470. doi: 10.1186/s12909-020-02391-z (PMC7691063; doi:10.1186/s12909-020-02391-z)
Supplement: Supplementary file 1 — Additional file 1. [file 12909_2020_2391_MOESM1_ESM.docx]

Think about what is behind the patient, family, and health problems

-Worksheet on the approach to social determinants of health

Consider the background factors that affect the health of patient (or family or community) whom you meet during this program. Find examples where healthcare professionals (if any) are addressing the factor.

You will be giving a presentation on the final day. Write about the case on this worksheet and bring it with you.

1. Please describe the patient and how you encountered that patient

２. Information gathered about background factors affecting that patient’s health and any other things that you noticed

3. (Of things you don’t know yet) factors that may be affecting that person’s health

4. Examples of addressing social determinants of health during the program, if any
