# Supplementary material for: Students’ understanding of social determinants of health in a community-based curriculum: a general inductive approach for qualitative data analysis
Source: BMC Med Educ. 2020 Nov 25;20:470. doi: 10.1186/s12909-020-02391-z (PMC7691063; doi:10.1186/s12909-020-02391-z)
Supplement: Supplementary file 2 — Additional file 2. [file 12909_2020_2391_MOESM2_ESM.docx]

4-week final report

1. Describe what you learned from the treatment of patients by healthcare professionals (not limited to physicians) in community settings, referring to the evaluation criteria and following the instructions below.

| From the specific responses of healthcare professionals to patients (who responded to whom and how?), describe 1) the significance of gathering information beyond the patient’s illness and 2) the significance of understanding the characteristics of the family and community. |
| --- |
|  |
| What role do you think healthcare professionals should play with respect to individuals (patients, local residents, etc.) in the community? |
|  |

2. Describe what you learned about social determinants of health as a healthcare professional (not limited to physicians), referring to the evaluation criteria and following the instructions below.

| From the cases you (or your colleagues) experienced, describe what you learned about the significance of healthcare providers being aware of the social determinants of health. |
| --- |
|  |
| What roles do you think healthcare professionals should play in the community to support the health of the community? |
|  |
