# Supplementary material for: Students’ understanding of social determinants of health in a community-based curriculum: a general inductive approach for qualitative data analysis
Source: BMC Med Educ. 2020 Nov 25;20:470. doi: 10.1186/s12909-020-02391-z (PMC7691063; doi:10.1186/s12909-020-02391-z)
Supplement: Supplementary file 3 — Additional file 3. [file 12909_2020_2391_MOESM3_ESM.docx]

**Rubric for grading the 4-week report on social determinants of health**

| **Theme** | **Evaluation criteria** | **Excellent（Ａ＋）** | **Good（Ａ）** | **Fair（Ｂ）** | **Needs**  **improvement（Ｃ）** | **Fail（D)** |
| --- | --- | --- | --- | --- | --- | --- |
| **Understanding**  **social determinants**  **of health** | 1. Concretely describes cases related to the social determinants of health. 2. Based on the above experience, describes the significance of being aware of the social determinants of health. 3. Describes in one’s own words the role that healthcare professionals (including non-physicians) should play in supporting the health of the community. | Understands the characteristics of each region based on multiple experiences in community settings and understands upstream societal problems from multiple perspectives. Understands the role of healthcare professionals from multiple perspectives based on interprofessional collaboration and the current status of the system and medical resources. | Integrates multiple experiences in community settings and sufficiently understands the patient in relation to multiple social determinants of health. Learns the role of healthcare professionals in the community from experiences. | Describes an experience in the community and the characteristics of the community in relation to social determinants of health. | Insufficient description of an experience in the community, or poor understanding of social determinants of health. | The requested content is not provided. |
